# Drug Resistance in Natural Isolates of *Leishmania donovani* s.l. Promastigotes Is Dependent of Pgp170 Expression

**DOI:** 10.1371/journal.pone.0065467

**Published:** 2013-06-11

**Authors:** Ippokratis Messaritakis, Vasiliki Christodoulou, Apostolos Mazeris, Eleni Koutala, Antonia Vlahou, Sevasti Papadogiorgaki, Maria Antoniou

**Affiliations:** 1 Laboratory of Clinical Bacteriology, Parasitology, Zoonoses and Geographical Medicine, Faculty of Medicine, University of Crete, Crete, Greece; 2 Veterinary Services of Cyprus, Nicosia, Cyprus; 3 Laboratory of Flow Cytometry, Faculty of Medicine, University of Crete, Crete, Greece; 4 Institute of Biomedical Research, Athens Academy, Athens, Greece; 5 Vassilis Galanopoulos Electron Microscope Facility, Biology Department, University of Crete, Crete, Greece; Albert Einstein College of Medicine, United States of America

## Abstract

Resistance of pathogens to drugs is a growing concern regarding many diseases. Parasites like *Leishmania, Plasmodium* and *Entamoeba histolytica;* and neoplastic cells, present the multidrug-resistant phenotype rendering chemotherapy ineffective. The acquired resistance of *Leishmania* to antimony has generated intense research on the mechanisms involved but the question has not yet been resolved. To test the hypothesis that drug efflux in *Leishmania*, as measured by flow cytometry using the fluorescent dye Rhodamine-123, is largely dependent on the number of efflux pumps an isolate can express, the amount of Pgp 170 molecules was assessed in ten field isolates (5 “resistant” and 5 “susceptible”) using: Western Blotting, Confocal and Transmission Electron Microscopy, and proteomics. Their survival after exposure to three antileishmanial drugs, *in vitro,* was evaluated and clinical data were compared to the *in vitro* results. All isolates were resistant to Glucantime but susceptible to Miltefosine, whilst Amphotericin B was more effective on the “susceptible” isolates. The MDR gene, expressing the transmembrane efflux pump Pgp 170, appears to play a key role in the phenomenon of drug resistance. When “susceptible” versus “resistant” parasites were compared, it was shown that the higher the number of Pgp 170 molecules the higher the Rhodamine-123 efflux from the parasite body and, when exposed to the drug, the number of efflux pumps increased. However, the rate of this increase was not linear and it is possible that there is a maximum number of Pgp 170 molecules an isolate can express. Nevertheless, the phenomenon is a complex one and other factors and proteins are involved in which the HSP-70 group proteins, detected in the “resistant” isolates, may play a significant role.

## Introduction

*Leishmania* parasites are transmitted by Phlebotomine sandflies causing leishmaniasis. *Leishmania donovani donovani* and *L. donovani infantum* are mainly responsible for visceral leishmaniasis (VL), the most severe form of the disease. In southern Europe VL is endemic due to *L. infantum* [1] but in the last years *L. donovani* has emerged in Cyprus both in the cutaneous and visceral form [2]. Whilst *L. infantum* is zoonotic, using the dog as reservoir host, *L. donovani* is considered anthroponotic; and as the two parasites meet in the host (vector and reservoir), there is danger of hybrid development with possible new characteristics, unfavourable to the patient [3]. Already, one dog examined in Cyprus, by K26 PCR [4], [5], was found to harbour both parasite species.

Control of the disease relies primarily on chemotherapy, in patients and dogs, but there is a limited number of drugs available, each with shortcomings [6]. Antimony-resistant *Leishmania* parasites have been reported from many endemic areas worldwide reaching epidemic proportions in the state of Bihar, India [7], [8]. This alarming situation intensified research into the mechanisms by which *Leishmania* acquires resistance to drugs. Drug resistance in this organism (but also in *Plasmodium* and *Entamoeba* parasites as well as in neoplastic cells) is associated with a multidrug-resistant (MDR) phenotype characterized by the over-expression of a P-glycoprotein, Pgp 170 (130 to 200 kDa) [7], [8], [9]. It acts as a transmembrane efflux pump for a diverse group of lipophilic compounds, including many chemically diverse drugs and fluorescent dyes as well as calcium channel blockers [10], [11]. The result of this pleiotropic effect is a reduced drug accumulation inside the cells and therefore the survival of the parasites or the MDR neoplastic cells [12]. The Pgp is a member of the super family binding cassette (ABC) transporters, responsible for transmembrane transport of a number of biological molecules and chemotherapeutic compounds [13]. More than 50 ABC transporters are known. About 15 have been characterized in human cells, two of which, PGP and MRP, are involved in MDR [13], [14], [15]. Understanding its role in *Leishmania*, would help in the development of a directed approach to chemotherapy for the successful treatment of leishmaniasis, and probably other parasitic diseases and cancers.

The hypothesis we tested was that drug efflux is dependent, largely, on the number of Pgp 170 molecules each isolate expresses. To investigate this hypothesis 70 strains, isolated from patients and dogs in Cyprus [5], were studied using Flow Cytometry (FCM) and the fluorescent dye Rhodamine-123 (Rhod-123), *in vitro*. The isolates were characterized as “resistant” and “susceptible” depending on the rate of efflux of Rhod-123, an established substrate for Pgp, from the parasite body. In order to relate the expression of Pgp 170 to the rate of Rhod-123 efflux, 10 isolates were chosen for further studies: 5 “resistant” and 5 “susceptible”. The amount of Pgp 170 molecules expressed by each of the 10 isolates, as untreated controls and after exposure to the antileishmanial drug Meglumine antimoniate, was compared by Confocal (CM) and Transmission Electron Microscopy (TEM) and by Western Blotting (WB) using the monospecific C219 monoclonal antibody that recognizes mammalian Pgp 170 [16]. To detect transmembrane proteins, and their expression in “resistant” and “susceptible” isolates, proteomic analysis was performed and the survival of the isolates after exposure to three antileishmanial drugs, *in vitro,* was evaluated and compared to clinical data.

## Materials and Methods

### Parasites

Seventy *Leishmania* strains isolated from patients (5, *L. donovani*) and dogs (65, *L. infantum*) during an epidemiological survey conducted in Cyprus [5], were used in Flow Cytometry (FCM) experiments to study the rate of efflux, of the fluorescent probe Rhod-123, from the parasite body. Freshly thawed parasites (exponential phase promastigotes) were used in all experiments. They were maintained in supplemented RPMI 1640 culture medium at 26±1°C [17], [18].

**Figure 1:**
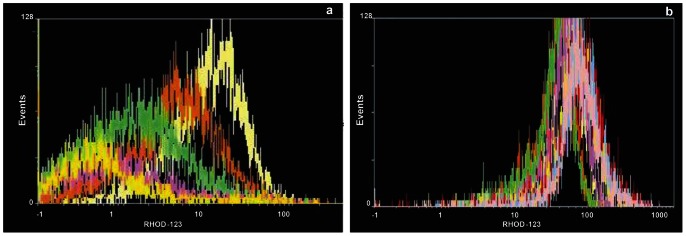
Rhod-123 efflux in *Leishmania* promastigotes observed by Flow Cytometry. High Rhod-123 efflux in the “resistant” dog isolate D5 (a). Low Rhod-123 efflux in the “susceptible” dog isolate D1 (b). Measurements were taken every 30 minutes, for two hours.

According to the FCM results [Christodoulou et al., unpublished data], 10 isolates were chosen for further studies: 5, with the highest Rhod-123 efflux rate (Figure 1a), classified as “resistant” (4 *L. infantum* and 1 *L. donovani*) and 5 with the lowest (Figure 1b), characterized as “susceptible” (4 *L. infantum* and 1 *L. donovani*) (Table 1).

**Table 1:** Mean Fluorescent Intensity (MFI) of the 10 Leishmania isolates measured by Flow Cytometry.

	Isolate Reference Code	Descriptive Code	Influx	efflux rate (slope “α”)	efflux rate (slope “β”)
**“susceptible” Isolates**	MCAN/CY/2005/CD45	D2	4.87	0.16	0.16
	MCAN/CY/2005/CD57	D7	6.90	0.87	0.64
	**MHOM/CY/2006/CH34**	**H1**	**20.53**	**1.36**	**0.22**
	MCAN/CY/2005/CD40	D6	15.17	1.55	0.92
	MCAN/CY/2006/CD87	D1	18.60	1.83	0.25
**“resistant” Isolates**	MCAN/CY/2006/CD83	D5	51.23	2.36	0.27
	MCAN/CY/2005/CD40	D8	37.27	2.88	0.68
	MCAN/CY/2006/CD85	D4	25.37	3.53	0.97
	MCAN/CY/2006/CD80	D3	60.25	4.26	2.48
	**MHOM/CY/2006/CH33**	**H2**	**30.83**	**6.00**	**0.50**

D1-D8: Dog isolates, *Leishmania infantum* MON-1; H1-H2: Human isolates, *Leishmania donovani* MON-37; Influx, MFI on baseline; Slope “α”, rate of efflux of Rhod-123 in 120 minutes; Slope “β”, rate of efflux of Rhod-123 in 120 minutes in the presence of Verapamil hydrochloride.

### THP-1 Cell Line: Culture and Infection

Freshly thawed cultures of the human monocytic cell line, THP-1 (Sigma-Aldrich, Inc., St Louis, MO, USA) were maintained in supplemented RPMI 1640 culture medium at 37°C, 5% CO_2_ and >80% humidity [18], [19]. They were infected with promastigotes of each of the 10 isolates at a ratio of 5 parasites: 1 host cell, in triplicates. After 3 hrs incubation the free promastigotes were removed using Histopaque 1077 (Sigma-Aldrich Inc., St Louis, MO, USA). THP-1 cells were washed, resuspended in 10 ml supplemented RPMI 1640 culture medium and incubated overnight at 37°C, 5% CO_2_ and >80% humidity.

### *In vitro* Acquired Resistance of Amastigotes

To investigate the possible effect of Meglumine antimoniate (Glucantime; Sanofi–Aventis, France) on the number of Pgp molecules in the intracellular stage of the parasite (the amastigote), 800 µg/ml Glucantime were added to the infected THP-1 cell cultures containing 5×10^5^ cells/ml. After incubation for 48 hrs, at 37°C, 5% CO_2_, >80% humidity, the cells were washed and the culture medium and Glucantime were replaced with fresh solution. The cells were further incubated for 12 hrs, washed and cytospin preparations were made using 100 µl from each culture. The preparations were fixed in ice-cold methanol for 10 min and double stained using immunofluorescence.

### Study of the Rate of Efflux of Rhod-123 in *Leishmania,* using FCM

The rate of efflux of the fluorescent probe Rhod-123 (Sigma-Aldrich Inc., St Louis, MO, USA) was tested in the 70 isolates using FCM (Beckman Coulter Epics Elite Flow Cytometer, equipped with argon - ion laser tuned to 630 nm). Promastigotes of each of the 70 isolates were washed and resuspended in 1 ml PBS, pH 7.2, containing 2×10^6^ parasites. Two groups, of two tubes each, were prepared with: a) 10^6^ parasites, b) 10^6^ parasites plus 10 µl Rhod-123 (diluted 1∶1600 in PBS, pH 7.2). PBS was added up to 500 µl in all tubes and they were incubated at 26±1°C, for 45 min, in a dark chamber. The parasites were washed, resuspended in 500 µl ice cold PBS, and 8 µl of the Pgp reversal agent Verapamil hydrochloride (Isoptin, Abbott Laboratories S.A., Greece) were added to the second tube of group b. From each of the four tubes, 100 µl were transferred into 4 RIA tubes and 3 µl Propidium Iodide (Sigma-Aldrich Inc., St Louis, MO, USA) were added, prior to each measurement, in 3 of the 4 tubes to exclude dead cells [17]. The mean fluorescence intensity (MFI) of 10.000 events was measured every 30 min, for 2 hrs, in triplicates, in order to follow the rate of Rhod-123 efflux with and without the effect of Verapamil hydrochloride for each isolate. Measurements were considered only if the percentage of dead parasites was <5%. Between measurements, the tubes were kept at 26±1°C, in a dark chamber. Data analysis was performed using the WinMDI 2.8 [J. Trotter (1993-1998)] software. Measuring MFI changes in time, in the promastigote stage, showed 6 out of the 70 isolates to have a high Rhod-123 efflux (Figure 1a) and were therefore characterized as “resistant” (Christodoulou et al., unpublished data).

### Detection of Pgp 170 Molecules, Using WB

Promastigotes of the 10 isolates (10^7^ parasites), in triplicates, were used. The parasites were washed in 0.9% NaCl, the pellet resuspended in 10 µl Urea Cracking Buffer (10 mM NaH_2_PO_4_, 1% SDS, 1% β-Mercaptoethanol, 5 M Urea, pH 7.0) and incubated at 37°C for 15 min. An equal volume of Urea - Laemmly buffer 2X (2 M TrisHCl pH 6.8, 10% SDS, 10% β-mercaptoethanol, 20% Glycerol, 0.2% Bromophenol Blue, 8 M Urea) was added, followed by a 15 min incubation at 37°C. The whole cell lysates were separated by 8% SDS-PAGE and electroblotted to 0.45 µm nitrocellulose membranes (Hoefer Scientific Instruments, USA) using a semi-dry transfer apparatus (Hoefer Scientific Instruments, USA) at 0.8 mA/cm^2^, for 1.5 hrs. The membranes were washed in 1X TBS (10X:50 mM Tris pH 8.0, 150 mM NaCl, 0.01% Triton) and incubated for 3 hrs at 4°C, under rotation, with the primary monoclonal anti-Pgp 170 C219 antibody (1∶50; antibody diluted in 2% blocking buffer - 2% (w/v) low-fat dried milk in 1X TBS; Alexis Biochemicals, Axxora, USA). For the visualization of the immunoreactive bands, two secondary antibodies with different conjugation capacities were used as follows: the membranes were washed and incubated for 1 hr at 4°C, under rotation, with the secondary antibody diluted in 5% blocking buffer as applicable: anti-human conjugated with Alkaline Phosphatase (DAKO, Denmark; dilution 1∶1000) or anti-rabbit HRP-conjugated (Jackson Immunoresearch Laboratories, Inc., USA; dilution 1∶10.000). The membranes were then washed at 4°C in 1X TBS and immunoreactive bands were visualized either: after incubation in Alkaline Phosphate buffer (100 mM NaCl, 1 M MgCl_2_, 100 mM Tris pH 9.5) for 10 min following incubation in NBT/BCIP (Pierce, USA) for 8 min at 4°C, or after incubation with enhanced chemiluminescence (ECL) (Supersignal West Pico Chemiluminescent Substrate, Thermo Scientific, Rockford, IL, USA). The emitted light was captured using a LumiImager cooled CCD camera (Boehringer Mannheim, Indianapolis, IN, USA).

### Double Immunofluorescence Staining of Intracellular Amastigotes, for CM

Fixed cytospin preparations of infected THP-1 cells were washed and permeabilized to allow intracellular antibody diffusion using 1% Triton (BDH Chemicals Ltd, Poole, UK). They were then washed and incubated with positive, against *Leishmania,* dog serum (for THP-1 cells infected with *L. infantum*) or human serum (for cells infected with *L. donovani*), for 30 min, at 37°C. After washing, the cells were further incubated with Fluorescein Isothyocianate (FITC) - labeled rabbit anti-dog IgG antibodies (Sigma-Aldrich Inc., St Louis, MO, USA) or anti-human IgG, Fluorescein Conj. F(ab’)_2_ (Sanofi Diagnostics Pasteur, France), respectively (dilution 1∶200), for 30 min, at 37°C, in a dark chamber. After washing, the cells were incubated for 2 hrs at room temperature, in a dark chamber, with the C219 monoclonal antibody (dilution 1∶50). They were washed and incubated for 45 min with the infrared Cy3 anti-mouse IgG secondary antibody (Jackson Immunoresearch Laboratories Inc., USA; dilution 1∶800). The slides were washed and analyzed under a Confocal Laser Scanning Microscope (Leica Microsystems GmbH, Germany) that uses argon/krypton, He/Ne and infrared lasers operating in multi-line mode, at excitation wavelengths of 488/568 nm, 633 nm and 561 nm, respectively. Images were processed using the Lite version of the Leica Confocal Software. Green and red fluorescence signals were related to the parasite body and the efflux pump, Pgp 170, respectively. Three preparations were assessed for each isolate and the Mean Fluorescent Intensity (MFI) was calculated.

### Immunogold Labeling of Pgp Molecules, for TEM

The most “resistant” (D3) and the most “susceptible” (D2) dog isolates were used in this study, as untreated controls and after incubation with Glucantime. THP-1 cells, infected with these isolates, were washed in Sodium Cacodylate Buffer (SCB; 0.1 M, pH 7.4) and the cell suspensions were incubated, for 30 min, in 1 ml SCB, pH 7.4, containing 2% paraformaldehyde (BDH Chemicals Ltd, Poole, UK) and 0.5% glutaraldehyde (BDH Chemicals Ltd, Poole, UK), for rapid penetration and fixation. The cells were then washed with SCB, pH 7.4, and 0.5 ml gelatin (Merck, Germany) was added. They were kept on ice for 10 min and dehydrated in 30%, 50% and 70% ethanol at 4°C. Infiltration was performed for 2 hrs using a mixture of 70% Ethanol/Resin (LR White, TAAB LAB) at a ratio of 1∶2. Cell embedding and polymerization was completed in gelatin capsules (LKB Bromma 2088 Ultratome V, Sweden).

Sections of the infected cells, placed on TEM-nickel grids, were incubated with 20 µl Blocking Buffer (1% BSA, 0.02% NaN_3_ in PBS, pH 7.5) for 30 min at room temperature. After washings (0.05% Tween, 0.1% BSA, 0.02% NaN_3_ in PBS, pH 7.5) the sections were incubated with 20 µl of the C219 monoclonal antibody, diluted 1∶50 in Ab solution (1% BSA, 0.02% NaN_3_ in PBS, pH 7.5) for 12 hrs at 4°C. The sections were rinsed in washing solution and incubated with 20 µl secondary antibody (Goat anti-mouse IgG 20 nm Gold, BB International, UK, diluted 1∶40 in Ab solution) for 2 hrs at room temperature. After washing, the grids were allowed to dry prior to TEM analysis (JEM-2100/Semi STEM/EDS, JEOL Ltd, Japan).

### Proteomic Analysis

Proteomic analysis was carried out on the promastigote stage of 4 representative isolates: 2 *L. donovani* (H1, H2) and 2 *L. infantum* (D2, D3) (H1, D2: were characterized as “susceptible” and H2, D3: as “resistant”) (Table 1). To extract and select the transmembrane proteins of the isolates, 10^8^ parasites of each isolate, in triplicates, were washed and membrane proteins were collected following sub cellular fractionation using the Qproteome Cell Compartment Kit (Qiagen, Valencia, CA, USA). The precipitates were resuspended in 200 µl rehydration buffer (7 M Urea, 2 M Thiourea, 4% CHAPS, 1% DTE, 2% IPG and 4% PI) and protein concentration was estimated using Bradford reagent (BioRad, UK). For the isoelectric focusing (IEF) 17 cm linear pH gradient IPG strips (ReadyStrip IPG Strip, BioRad, UK) were used. The strips were rehydrated with 200 µl rehydration buffer including 1.6 mg resuspended membrane cell protein. IEF followed for 112 kVh (250 V for 30 min, 5000 V for 28 hrs and 500 V for 10 hrs). The strips were sequentially equilibrated for 15 min each, in 100 µl rehydration buffer supplemented with 0.5% (w/v) DDT and 4.32% (w/v) iodoacetamide, respectively. Equilibrated IPG strips were separated across 12% SDS-PAGE gels [30% (w/v) acrylamide/PDA (Piperazine D-Acrylamide) (37.5∶ 1)] using a vertical system (Ettan DALTtwelve System Separation Unit, Amersham Biosciences, UK) and standard Tris/Glycine/SDS buffer. Gels were run at 75 mA/gel, at 18°C, stained with Coomassie Colloidal Blue Stain (Novex, Invitrogen, Carlsbad, CA) and Silver Nitrate Stain (SilverXpress Silver Staining Kit, Invitrogen, Carlsbad, CA), and scanned at a GS-800 imaging densitometer (BioRad, UK) in transmission mode. Gel images were analyzed using the PD Quest 6.1 software (BioRad, UK). A unique identification number was assigned to each matching spot on the different gels. Differential expression was defined based on t- test (p<0.05). Protein spots of interest were excised manually from the gels. Digestion with Porcine Trypsin (Promega, Southampton, UK) and peptide mass fingerprinting (PMF) was performed [20]. The peptide concentrate obtained was mixed with an equal volume of saturated solution of α-cyano-4-hydroxycinnamic in 1∶2 (v/v) with solution of 50% acetonitrile and 0.1% trifluoroacetic acid, and spotted directly on the target plate. Peptide masses were determined by MALDI-TOF-MS (Ultraflex, Bruker Daltonics, Bremen, Germany) and protein identification by MASCOT (Matrix-Science); searching in Leishpep, a protein database generated by annotation of *L. major* Friedlin sequence information [21]. The search criteria used were: carboxamidomethylation of cysteine residues (fixed modification), partial methionine oxidation (variable modification) and mass deviation smaller than 60 ppm. Using these settings, a MASCOT score of greater than 51 was considered significant (p<0.05).

### Drug Sensitivity Assays

The survival of promastigotes of the 10 isolates was estimated after their exposure to: Glucantime, Ambisome (Amphotericin B; Bristol-Myers Squibb, France) and Miltefosine (Hexadecylphosphocholine; Virbac, France). Promastigotes were incubated in 5 ml RPMI 1640 culture medium, in triplicates, at 26±1°C at a starting concentration of 10^6^ parasites/ml. The drug was added to the culture medium, after two-fold serial dilutions, starting from 50 µg/ml for Glucantime, 0.75 µg/ml for Ambisome, and 1.25 µg/ml for Miltefosine: 5 different concentrations and a control (no drug), per isolate. The cultures were maintained in sterile 6-well plates and the culture medium was replaced every 2 days (with fresh drug dilutions where appropriate). The parasite density was determined, every day, for 6 days, using a haemocytometer and Trypan blue (Sigma-Aldrich, Inc., St Louis, MO, USA) in order to exclude dead parasites.

### Ethical Considerations

Collection of the isolates used in this study has been described previously [5]. In all experimental trials the samples used were anonymized. The experiments and procedures described have been approved by the Institutional Animal Care and Use Committee of the University of Crete Medical School and conform with the European Union Directive 2010/63/EU regarding use of animals and biological specimens in research, as well as the relevant Hellenic legislation (Presidential Decree 160/91, under the Code Numbers 31 EE 05, 31 ΕΠΡ 04 and 31ΕΠ 020). Written informed consent was obtained from the patients involved and the dog owners, according to the aforementioned national legislations.

## Results

### Analysis by FCM

**Figure 2:**
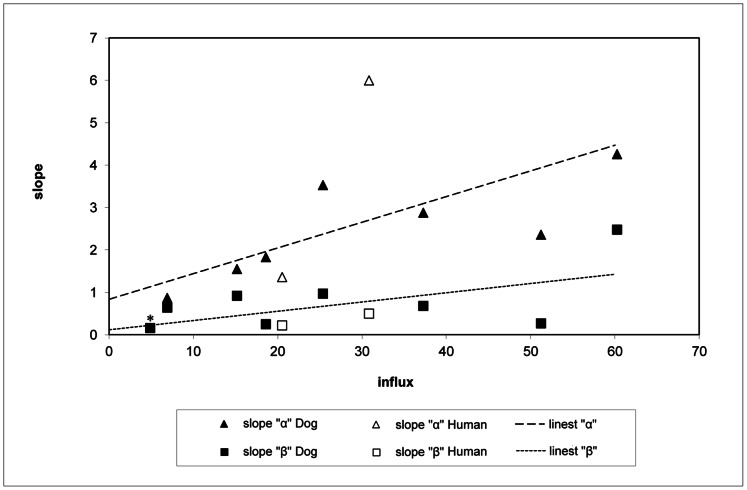
Rate of efflux of Rhod-123, in the 10 *Leishmania* isolates, estimated by Flow Cytometry. Efflux, both in the absence (slope “α”) and presence (slope “β”) of Verapamil hydrochloride was found to be correlated to the influx (the amount of drug that entered the parasite body: Mean Fluorescent Intensity on baseline (MFI) (c = 0.63 and c = 0.58, respectively). Efflux was blocked by Verapamil hydrochloride in all isolates except in isolate D2, with the lowest slope “α”, for which slope “β” had the same value (0.16)*.

Flow cytometry assays showed the rate of Rhod-123 efflux (slope “α”) of the 70 isolates to vary from 0.16 to 6.0 (Christodoulou et al., unpublished data). The highest rate of Rhod-123 efflux (slope “α” = 6.0) was presented by H2, a strain isolated from a 10 month-old girl from the Roma community of Cyprus with VL symptoms [22]. The higher the influx (MFI on baseline), the higher was slope “α” and, to a lesser degree, the higher the rate of efflux of Rhod-123 in the presence of the reversal agent Verapamil hydrochloride (slope “β”) (Figure 2) (Table 1).

### Analysis by WB

**Figure 3:**
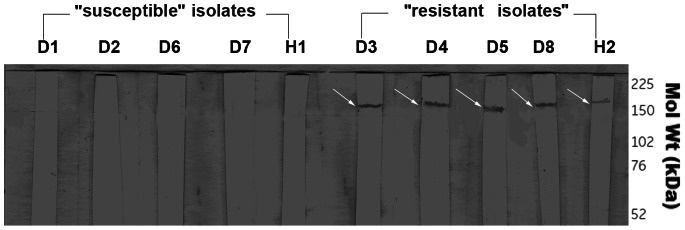
Detection of Pgp 170 by Western Blotting in the 10 *Leishmania* isolates. The Pgp 170 molecules (130-200 kDa) were evident in the “resistant” (high Rhod-123 efflux) but not in the “susceptible” (low Rhod-123 efflux) isolates. The “resistant” human isolate (H2) presented lower signal compared to the dog “resistant” isolates. For the evaluation of the Pgp expression in each isolate, the C219 monoclonal antibody and exponential phase promastigotes, at a concentration of 10^7^ parasites, were used.

The P-glycoprotein hybridization signal was amplified only in the 5 isolates characterized by FCM as “resistant”. The signal obtained was stronger in the *L. infantum* isolates compared to the *L. donovani* isolate (Figure 3).

### Analysis by CM

**Figure 4:**
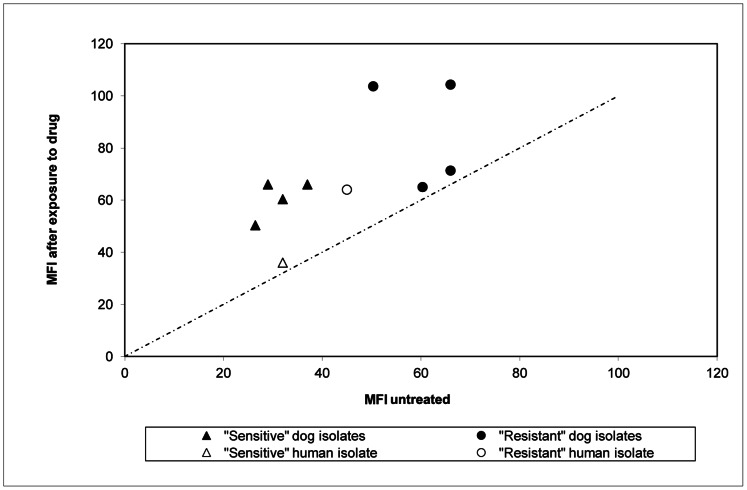
Treatment with Glucantime resulted in an increase in the number of Pgp molecules. Mean Fluorescent Intensity (MFI) of the 10 *Leishmania* isolates, measured by Flow Cytometry, showed an increase in the number of Pgp molecules, in all isolates, if they had previously been treated with the drug Glucantime compared to the untreated controls. This increase was not linear (r_2_ = 0.27).

**Figure 5:**
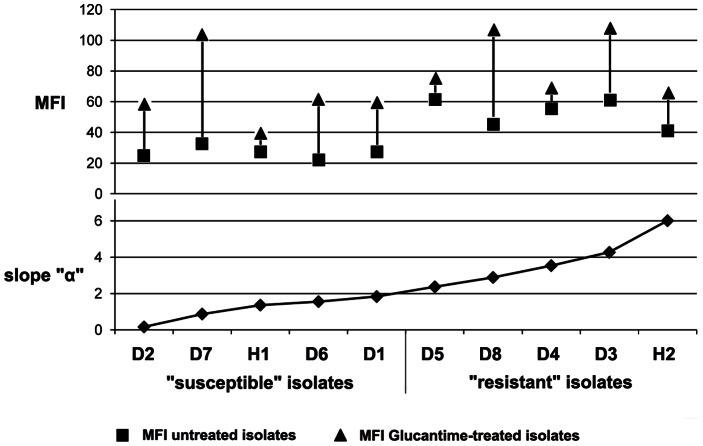
Mean Fluorescent Intensity (MFI) before and after treatment with Glucantime. In all 10 isolates, irrespective of the rate of Rhod-123 efflux (slope “α”), treatment with Glucantime resulted in an increase of MFI, estimated by Confocal Microscopy (CM), indicating an increase in the number of Pgp 170 molecules.

**Figure 6:**
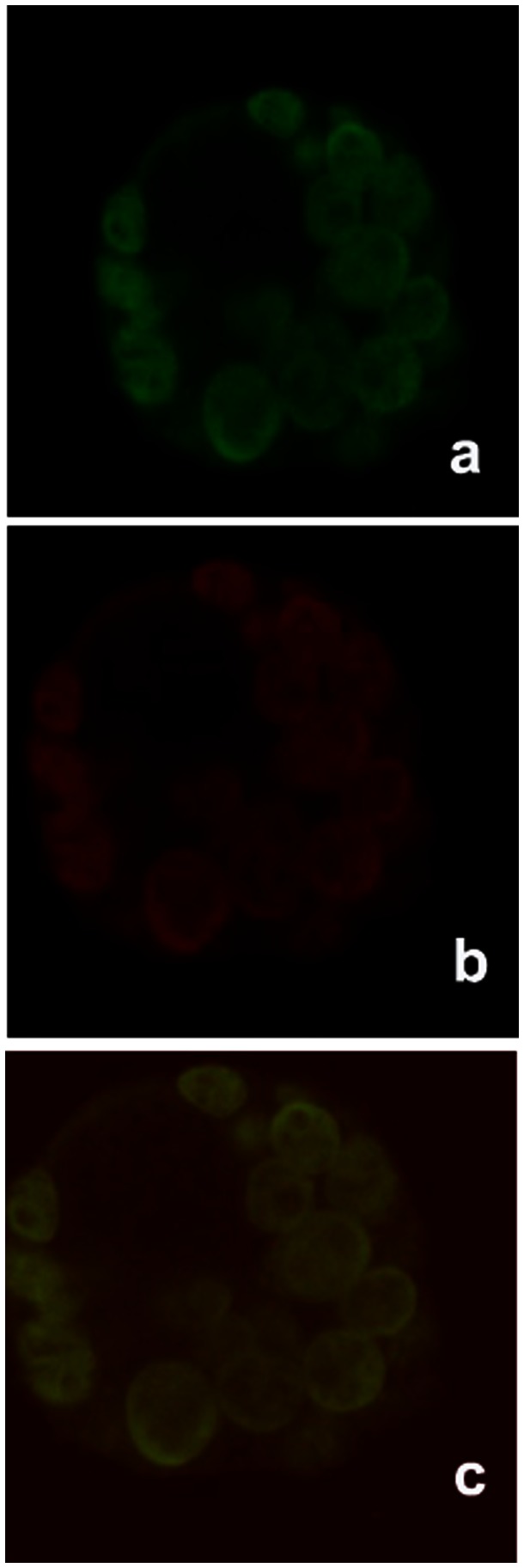
Location of Pgp molecules in the body of *Leishmania* observed under Confocal Microscopy. *Leishmania* amastigotes (inside THP-1 infected cells) marked with double immunofluorescence: positive dog serum and FITC (green signal) (a); Pgp 170 molecules marked with the C219 monoclonal antibody and the infrared Cy3 anti-mouse IgG secondary antibody (red signal) (b); overlay of image a and b (c). Image c confirms the location of Pgp 170 molecules on the membrane of the parasite body.

Mean Fluorescent Intensity (MFI) of Pgp 170 was stronger in the 5 “resistant” compared to the 5 “susceptible” isolates. In all isolates, irrespective of the rate of Rhod-123 efflux (slope “α”), treatment with Glucantime resulted in an increase of MFI (paired, single tail t-test, p<0.002), indicating an increase in the number of Pgp 170 molecules. This increase was not linear (r2 = 0.27) and was higher in the “susceptible” compared to the “resistant” isolates (p<0.08) (Figures 4,5). After double immunofluorescence, overlaying the green (positive for *Leishmania* serum and FITC) and red (C219 monoclonal antibody and infrared Cy3 anti-mouse IgG) signals, confirmed the location of Pgp 170 on the membrane of the parasite body inside the THP-1 cell (Figure 6: a,b,c respectively).

### Analysis by TEM

**Figure 7:**
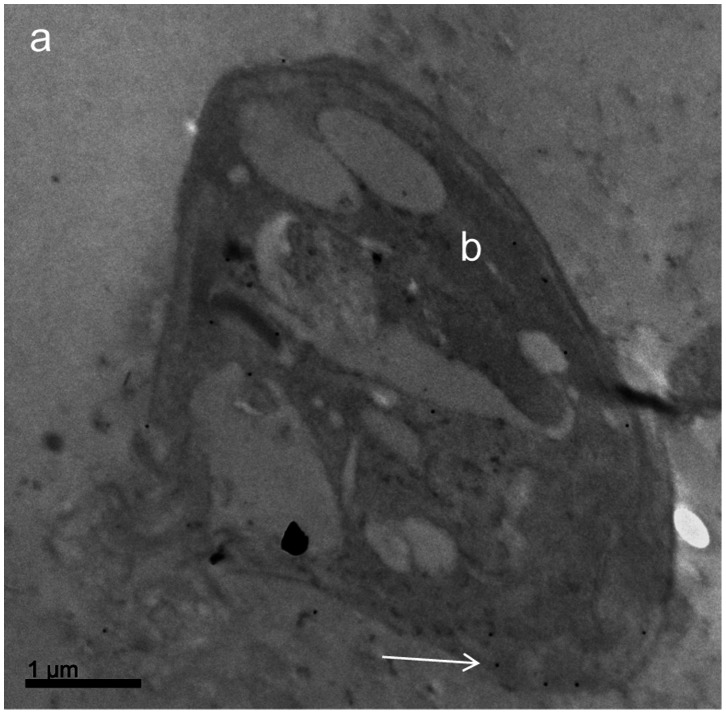
Detection of Pgp 170 molecules, by Transmission Electron Microscopy, after immunogold labeling. *Leishmania* amastigote (inside a THP-1 infected cell): cytoplasm of the THP-1 cell (a); *Leishmania infantum* body (b). Black spots (as indicated by white arrow) show Pgp 170 molecules (gold granules after immunogold labeling using the C219 monoclonal antibody).

**Figure 8:**
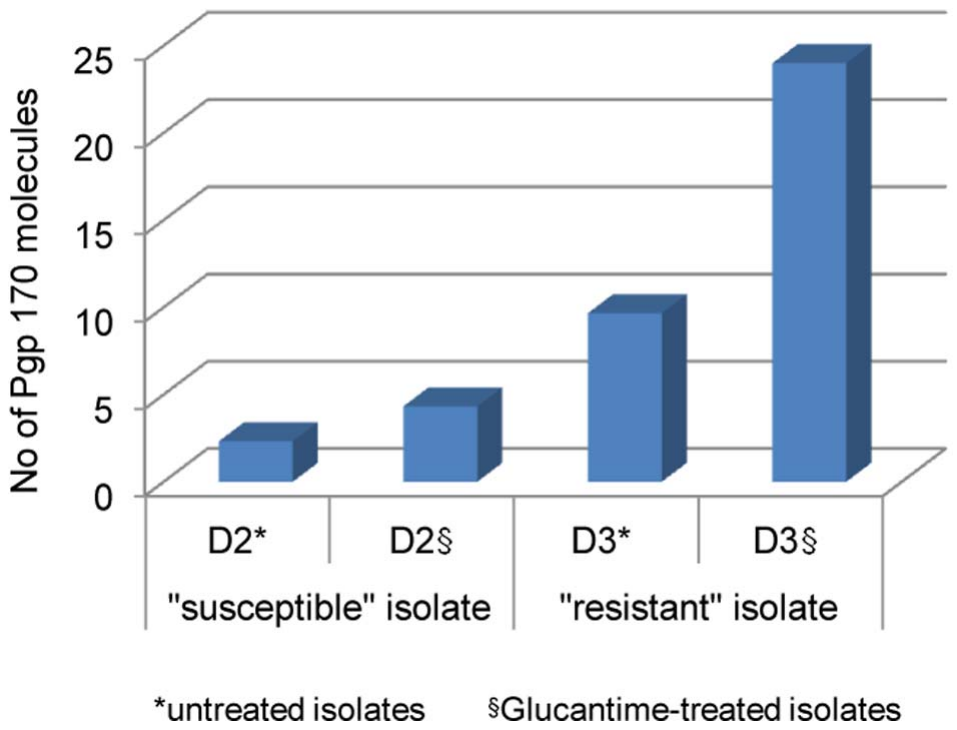
Number of Pgp molecules in a ''resistant'' and a ''susceptible'' isolate before/after exposure to Glucantime. The number of Pgp 170 molecules was found in higher numbers in the “resistant” compared to the “susceptible” isolate. This number increased, in both isolates, after exposure to Glucantime.

The Pgp 170 molecules were found mainly on the cellular membrane of the parasite (Figure 7) and in small numbers on intracellular membranes of organelles. The number of the Pgp molecules was higher in the “resistant” compared to the “susceptible” isolate and this number increased, in both isolates, if they had been incubated with meglumine antimoniate (Figure 8).

### Proteomic Analysis

After post staining with Coomassie Brilliant Blue, the gels revealed only over-expressed proteins or proteins of high molecular weight. Staining with Silver Nitrate, however, revealed 300 and 304 protein spots for each of the two “susceptible” and “resistant” isolates, respectively. Of all the 1208 protein spots, 6 appeared to represent significantly different proteins or protein expressions between the two groups. Of those, 4 were detected only in the “resistant” isolates and 2 in both groups but were over-expressed in the “resistant” isolates (p<0.05) (Table 2). Using mass spectrometry, only the 4 proteins detected in the “resistant” isolates were identified as: Heat Shock 70 kDa protein (HSP-70) in isolate H2 (*L. donovani*), HSP-70 - related mitochondrial precursor (2 spots) and putative uncharacterized protein (∼40 kDa) in isolate D3 (*L. infantum*) (Table 2).

**Table 2:** Proteins detected by proteomic analysis and Silver Nitrate Staining.

Title	Isolate code and spot number (SSP)	Mascot Score	MS Coverage	Protein MW	Method	Accession	pI-Value
Heat shock 70 kDa protein - *L. donovani*	H2/4719	108	27	71410	Tryptic Mass MALDI TOF	HSP70_LEIDO	5,2
Heat shock 70 kDa - related protein 1, mitochondrial precursor –*L. infantum*	D3/3719	207	47	68915	Tryptic Mass MALDI TOF	HSP71_LEIMA	5,4
Heat shock 70 kDa - related protein 1, mitochondrial precursor –*L. infantum*	D3/4716	154	35	68915	Tryptic Mass MALDI TOF	HSP71_LEIMA	5,4
Putative uncharacterized protein - *L. infantum*	D3/5218	135	56	36576	Tryptic Mass MALDI TOF	A4HXA8_LEIIN	5,6

Of the 1208 protein spots detected, 6 appeared to represent significantly different proteins between the two groups of parasites. Of those, 4 were detected only in the “resistant” isolates (H2, D3) and 2 in both groups but over-expressed in the “resistant” isolates (p<0.05).

### *In vitro* Drug Sensitivity of the 10 Isolates

**Figure 9:**
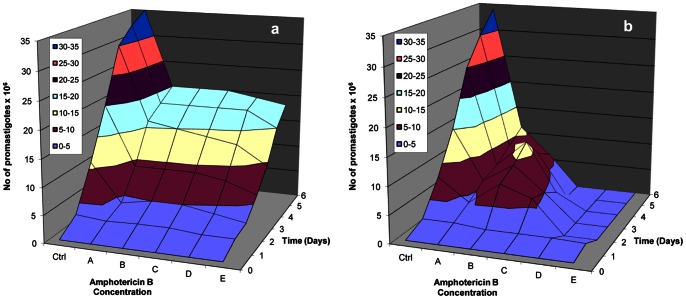
Survival of *Leishmania* promastigotes in different Amphotericin B concentrations, in time. Survival of the “resistant” dog isolate D5 (a) and of the “susceptible” dog isolate D1 (b), in different drug concentrations for 6 days.

The *in vitro* assay, testing resistance of the isolates to Glucantime (SbV), showed all 10 isolates requiring the maximum dose of 800 µg/ml, for 4 to 6 days, in order for population growth to be inhibited by half (IC_50_); no discrimination between “susceptible” and “resistant” isolates was observed. It has been shown that SbV works after its reduction to SbIII [23]. In preliminary experiments, using promastigotes, it was found that a higher dose of SbV was required for IC_50_ in the 10 isolates compared to SbIII, (results not shown). Amphotericin B led to a gradual reduction of the promastigote population of all 10 isolates, with a more severe effect on the “susceptible” isolates (Figure 9); none of the populations was eliminated by 100% before day 6 of the experiment. All 10 isolates were “susceptible” in all Miltefosine concentrations used, from the first day of the experiment.

## Discussion

Resistance of pathogens to drugs is an escalating phenomenon in many diseases rendering chemotherapy ineffective. Multidrug resistance commonly takes place via the overproduction of ATP-binding cassette (ABC) transporter proteins which act as broad specific drug efflux pumps. Apart from *Leishmania,* the protozoan parasites *Plasmodium,* causing malaria [24] and *Entamoeba histolytica*, responsible for human amoebiasis [25], present the MDR phenotype.

Two ABC transporters are implicated in *Leishmania* drug resistance. The PGPA, involved in arsenic and antimony compounds, and the MDR [10]. It has been shown that Leishmania, under arsenite and methotrexate selection, overamplifies and expresses genes (e.g. ItpgpA) that are part of the gene family encoding the Pgp [26], [27], [28], [29]. A member of this gene family (ldmdr1) can induce a drug-resistant phenotype in *Leishmania* [30] and has been molecularly characterized in *L. donovani* [31]. The amino acid sequence of Pgp derived from *L. donovani* (GenBank: AAA02977.1, [31]), after performing Protein BLAST [32] indicated 93% identity with the MDR1 protein of *Ovis aries* (GenBank: CAM33439.1) and 91% identity with the MDR1 protein of *Homo sapiens* (GenBank: AAA59576.1) suggesting significant homology with the mammalian Pgp.

The efflux rate of Rhod-123, measured by FCM, indicates the ability of the isolate to actively and selectively expel the drug from its body (Figure 1: a), thus escaping death [10], [33]. It is expected that the higher the pump activity, the lesser MFI inside the parasite body and this rate can be measured, by the Rhod-123 accumulation assay, as the loss of MFI from the body of the isolate in time (Figure 1: a, b), (slope “α”). This rate may depend on the number, possibly on the effectiveness/functionality of the Pgp 170 efflux pumps each isolate can express [34], but also on other proteins.

In this study, the expression of the efflux pump, Pgp 170, was evaluated in field isolates of *L. donovani* and *L. infantum,* from Cyprus and related to drug resistance. Slope “α” was found to be different in the 70 *Leishmania* isolates tested, ranging from 0.16 to 6.0 (Christodoulou et al., unpublished data). Using Verapamil hydrochloride, blocker of MDR [34], the specificity of the pumps was checked. Efflux appeared to be blocked by Verapamil hydrochloride in the 70 isolates (p<0.0001), but to a different degree (Table 1). Influx (the amount of drug that entered the parasite body: MFI on baseline) was found to be correlated to the rate of efflux, both in the absence and presence of Verapamil hydrochloride in the 70 isolates (p<0.0001 in both cases) (Christodoulou et al., unpublished data). Working on Indian field isolates of *L. donovani,* it was suggested that antimony resistance was due to a MRP-like pump; and since the blockers Verapamil hydrochloride, probenecid and ofloxacin failed to enhance Rhod-123 fluorescence, it was concluded that this was an indication of the absence of a classical MDR pump in these isolates [34]. Although the Cyprus *L. donovani* strains have, like the Indian strains, zymodeme profile MON-37 [2], [35] they were found to be genetically different [36]. Further studies are required to explain the differences observed in the *L. donovani* populations from the two countries regarding drug resistance.

The final amount of drug remaining in the parasite body is the decisive factor for the survival of the parasite during chemotherapy. This amount is the consequence of the amount of drug entering (influx) and the rate at which it is expelled from the body (efflux; slope “α”). These factors are interrelated by chemical and physical laws and Pgp 170, as well as other proteins, appears to be involved. To investigate the expression levels of Pgp 170 in *Leishmania* the C219 monoclonal antibody, raised against CHO Pgp (Pgp 1 or ABCB1) (Product datasheet, Alexis Biochemicals, Axxora, USA) was used. The number of Pgp 170 efflux pumps, assessed quantitatively (by TEM) and semi-quantitatively (by CM and WB) was consistently found to be higher in the isolates with a higher rate of efflux, as shown by FCM (Figures 8, 4, 5, 3 respectively). The signal detected by WB and CM accounts for all Pgp molecules: found on the membrane of the parasite body, where they are predominantly located and, in small numbers found on intracellular membranes of organelles, most probably where they are produced (Figure 7). This number is related to the number of MDR genes the isolate has; which, when activated, expresses more Pgp molecules [37] thus providing the isolate with acquired resistance. The relationship between Pgp overexpression and the MDR-1 gene has been shown in DNA transfection studies, in *Leishmania*, and related to drug resistance [38]. Interestingly, 65% of the Mediterranean *Leishmania* strains have been shown to have amplified MDR -1 genes [37].

Our results are in agreement with the results of previous studies showing that a *Leishmania* isolate may become resistant after exposure to a drug, *in vitro* and *in vivo* [39]. However, in the assays performed, the increase in Pgp 170 molecules after exposure to Glucantime was not linear and each isolate showed a different capacity for this increase. Three out of the 10 isolates studied appeared to have reached the maximum of their potential *in vitro,* expressing a low increase in MFI after exposure to the drug: the “susceptible” human isolate (H1) and two “resistant” dog isolates (D4 and D5) (Figure 5).

Previous studies [40], [41], [42], demonstrated that the drug efflux pump MRPA shows species variation. For this reason we used two species in this investigation: *L. infantum* and *L. donovani* from the island of Cyprus. Each isolate’s population members are not clones and may behave differently [43], but the isolate’s overall behaviour will determine the outcome of chemotherapy. In addition, a mixed infection may be present in a patient with two or more species/subspecies of the parasite, presenting a different degree of drug resistance each, further complicating chemotherapy [44].

When the results of the 2 human isolates (*L. donovani*) were related to the 8 dog isolates (*L. infantum*), interesting points arose. The human isolate H2 presented the highest Rhod-123 efflux rate by FCM (slope “α” = 6.0) yet, although Pgp molecules were detected by WB, the intensity of the signal was lower than that of the “resistant” isolates of dog origin (Figure 3). This finding was consistent with the MFI reading by CM which, again, was lower than that of the dog “resistant” isolates (Figure 5), indicating that other factors may also be involved in drug resistance in the two *Leishmania* spp. Strain H2 was isolated from a 10 month-old VL patient who required two treatments with liposomal Amphotericine B before cure was achieved [22]. Can the high resistance of isolate H2, *in vivo* and *in vitro* possessing a relatively lower number of efflux pumps, be explained by the presence of the HSP-70 protein?

It has been shown that antimony resistance is a feature of the parasite conserved in both stages in its life cycle, the promastigote and the amastigote [34], and that the proteome remains at 90–94% undifferentiated throughout the life of the parasite [45]. Proteomic analysis, carried out on the promastigote stage, detected an HSP-70 protein in the *L. donovani*, “resistant” human isolate (H2, slope “α” = 6.0) and an HSP-70 related protein in the *L. infantum* (dog isolate) with the highest Rhod-123 efflux (D3, slope “α” = 4.26; Table 2), indicating that the isolates express different levels of HSP. Is this differential expression related to drug resistance? It has been demonstrated that complexes between HSP-70 and co-chaperones have specific functions, including roles in pro-folding, pro-degradation and pro-trafficking pathways [46] and that HSP-70 and HSP-70 related proteins translocate into the plasma membrane of cells and parasites, like *Leishmania*, under stress conditions [47], [48]. Similar research on resistant *L. donovani* isolates led to the identification of heat shock proteins (HSP-70 and HSP-83), a small calpain-related protein [49], [50], histones (H1, H2A and H4) and MAP kinase 1 [51]. It appears that an isolate possessing heat shock proteins, with relatively low Pgp 170 expression, can have a high Rhod-123 efflux rate. The differential expression of HSP-70 in isolates with different efflux rates needs to be further investigated before and after treatment of the parasites with HSP-70 inhibitors in order to verify and quantify this translocation [52], [53]. It is evident that understanding HSP-70 biology can open a new window into the efforts for the development of novel therapeutic drugs [46]. In the 4 isolates, 2 unidentified proteins were detected but in lower concentration in the “susceptible” compared to the “resistant” isolates (Table 2). Proteomic analysis, comparing untreated and Glucantime treated parasites, is necessary before definite conclusions can be drawn on the presence and role of other molecular components involved in drug resistance.

We know that *Leishmania’ s* resistance to Glucantime, after 60 years of use, has become a serious problem in combating the disease worldwide and there are areas where it can no longer be used for effective treatment. Yet, the mode of action of antimony is still poorly understood [6]. All 10 isolates tested *in vitro* indicated non responsiveness to Glucantime, allowing the survival of “susceptible” and “resistant” isolates at a concentration of 800 µg/ml (dose used for treating visceral leishmaniasis patients). Exposure to Amphotericine B, a drug for which resistance is not known [54], revealed 2 “resistant” isolates requiring a higher dose of the drug (D5 and D8) (Figure 9). The human isolate, H2, which exhibited resistance *in vivo* [22], required 4 days at a concentration of 3 µg/ml for IC_50._ The new drug, Miltefosine, the first antileishmanial drug administered *per os* was able to kill all 10 isolates at the lowest concentration tested after one to two days of exposure. This is a valuable drug and should be used with caution to avoid the development of resistance.

Multidrug resistance is a complex phenomenon depending on genetic, parasite and host factors as well as environmental conditions. Resistance, to be confronted, must be clearly understood. No molecular markers of resistance are yet available and a simple laboratory method, to distinguish “resistant” from “susceptible” isolates, is necessary in order to decide on treatment to avoid relapses and resistance development. Rhod-123 is increasingly used as a tracer dye for MDR studies and the optimal parameters for its active and passive uptake into cells have been characterised [34]. As the disease is spreading, with the WHO leishmaniasis control team reporting 96 endemic countries today [55], continuous surveillance must be maintained to monitor the risk from the ongoing emergence and spread of drug resistance in this parasite which poses a threat to public health. All evidence indicates that resistance to drugs is a phenomenon that can be acquired *in vitro* and *in vivo*; the question is whether the acquired resistance is transmitted, fully or partly, from host to host via the sandfly vector.
